# ERGA-BGE Chromosome-Level Genome of *Patella rustica* Linnaeus, 1758: a Resource to Investigate Responses to Global Warming in the intertidal

**DOI:** 10.12688/openreseurope.21608.2

**Published:** 2026-03-09

**Authors:** Rui Faria, Rocío Nieto Vilela, Fernando P Lima, Rita Monteiro, Astrid Böhne, Thomas Marcussen, Torsten H Struck, Rebekah A Oomen, Marta Gut, Laura Aguilera, Tyler Alioto, Francisco Câmara Ferreira, Jèssica Gómez-Garrido, Fernando Cruz, Anna Lazar, Leanne Haggerty, Fergal Martin, Tom Brown

**Affiliations:** 1 CIBIO, InBIO Laboratório Associado, Universidade do Porto Centro de Investigacao em Biodiversidade e Recursos Geneticos, Vairão, Porto, Portugal; 2 BIOPOLIS Program in Genomics, Biodiversity and Land Planning, CIBIO, Universidade do Porto, Vairão, Portugal; 3 Departamento de Biologia, Faculdade de Ciências, Universidade do Porto, Porto, Portugal; 4 Leibniz Institute for the Analysis of Biodiversity Change - Museum Koenig Bonn, Bonn, Germany; 5 Natural History Museum, University of Oslo, Oslo, Norway; 6 University of Oslo Centre for Ecological and Evolutionary Synthesis, Oslo, Norway; 7 Department of Biological Sciences, University of New Brunswick Saint John, Saint John, New Brunswick, Canada; 8 Tjärnö Marine Laboratory, University of Gothenburg, Gothenburg, Sweden; 9 Centre for Coastal Research, University of Agder, Kristiansand, Norway; 10 Centro Nacional de Análisis Genómico (CNAG), Barcelona, Spain; 11 Universitat de Barcelona (UB), Barcelona, Spain; 12 European Molecular Biology Laboratory, European Bioinformatics Institute, Hinxton, UK; 13 Leibniz Institute for Zoo and Wildlife Research, Berlin, Germany; 14 BeGenDiv, Berlin Center for Genomics in Biodiversity Research, Berlin, Germany

**Keywords:** Patella rustica, genome assembly, European Reference Genome Atlas, Biodiversity Genomics Europe, Earth Biogenome Project, Lusitanian limpet, North-eastern Atlantic, Mediterranean Sea, Sentinels, Thermal stress, Evolutionary studies

## Abstract

The reference genome of *Patella rustica* Linnaeus, 1758 (Gastropoda, Patellidae) will offer a unique opportunity to understand how intertidal organisms respond to the effects of global warming, contributing to improve our predictions about the impact of climate change on the maintenance and diversification of marine biodiversity. A total of 9 contiguous chromosomal pseudomolecules were assembled from the genome sequence, made up from Oxford Nanopore Technologies (ONT) long-reads, WGS and Chromatin-Conformation-Capture (Hi-C) short-read sequences. This chromosome-level assembly encompasses 719 Mb, composed of 157 contigs and 24 scaffolds, with contig and scaffold N50 values of 8.52 Mb and 83.6 Mb, respectively, and assembly and annotation BUSCO completeness of 97.7% and 96.5%, respectively. The production of this genome assembly and annotation was coordinated and supported by the European Reference Genome Atlas (ERGA) and Biodiversity Genomics Europe project (BGE).

## Introduction

A constantly changing environment challenges organisms to adapt and survive ( Hoffman and Sgrò, 2011). This is particularly true for marine organisms living in the intertidal zone, where they must cope with regular environmental fluctuations caused by tides, as well as the increasing pressure from human-caused global warming ( Helmuth et al., 2006; Somero, 2010). Gaining knowledge on how intertidal organisms cope with environmental stress, adapt, and continue to diversify is therefore urgent in the context of the current climate crisis ( Trégarot et al., 2024) and the pressing need for effective conservation efforts.

Limpets are among the most common marine gastropods inhabiting the rocky intertidal zones worldwide ( Firth, 2021). Of these, species of the genus *Patella*, which are distributed along the Northeastern Atlantic and Mediterranean coasts, have been extensively studied in relation to environmental stress, among other species ( *Siphonaria* sp., Teske et al., 2011; barnacles and topshells, Broitman et al., 2008; for more invertebrates, see Sokolova et al., 2012). These species can serve as sentinels, providing insights into how marine biodiversity responds to climate change and the health of coastal ecosystems ( Hawkins et al., 2009). Consequently, they can help improve predictions of the impacts of global warming on marine communities and ecosystems.

**Figure 1.**
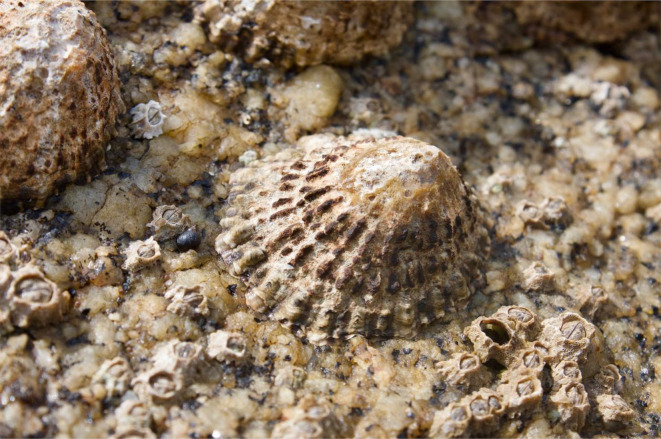
Example image of *Patella rustica.* Image taken by Fernando P. Lima.

This is especially true for *Patella rustica*, also known as the Lusitanian limpet, which is found throughout the Mediterranean basin and along the northeastern Atlantic coast from Southern France (Biarritz) to Mauritania, as well as in the Macaronesian archipelagos, excluding the Azores ( Fischer-Piètte, 1955; Crisp and Fischer-Piètte, 1959; Christiaens, 1973; Weber and Hawkins, 2005). It can be easily distinguished from other *Patella* species by the presence of brown spots on the upper part of the shell ( Figure 1). Several studies have documented recent changes in its distribution, particularly the bridging of a historical distribution gap in northwestern Iberia ( Fischer-Piètte and Gaillard, 1959; Lima et al., 2006). This distribution change was probably driven by rising sea temperatures, increased river runoff, and decreased upwelling intensity off the Portuguese coast ( Lima et al., 2006, 2007; Sousa et al., 2012). While genetic diversity has remained stable in colonizing populations ( Ribeiro et al., 2010), reliance solely on conventional markers (mtDNA and microsatellites) has limited understanding of both demographic history and adaptive genomic features. Addressing this gap is crucial for predicting further northward expansions and assessing the species’ impact on local communities and ecosystems.

It is now increasingly recognized that public genomic resources, specifically high-quality reference genomes, can be highly valuable for biodiversity monitoring and can support conservation efforts by providing essential information to guide management decisions ( Theissinger et al., 2023). Additionally, they offer a crucial foundation for assessing how species adapt to drastic environmental changes and further diversify amid global warming ( Formenti et al., 2022). While we are beginning to better understand these evolutionary processes in certain marine species with low dispersal (e.g., *Littorina* marine snails, Faria et al., 2019, 2021; Morales et al., 2019; Westram et al., 2018), we are still far from understanding how speciation occurs in most marine species, particularly those with high dispersal, where gene flow can oppose divergent selection and reproductive isolation ( Potkamp and Fransen, 2019; Barry et al., 2024).

Here, we present a chromosome-level genome of *Patella rustica*, to support intertidal biodiversity monitoring amid climate change. Along with recent chromosome-level reference genomes of five other *Patella* species (GCA_964341155.2, Hawkins et al., 2023, 2024, Mieszkowska et al., 2025; Lawniczak, 2022), this will help uncover insights into their evolutionary history and investigate the development of reproductive barriers within this genus ( Sá Pinto et al., 2010), including the role of structural variants in diversification ( Petraccioli et al., 2010).

The creation of this reference genome results from a collaborative effort coordinated by the European Reference Genome Atlas (ERGA) within the Biodiversity Genomics Europe (BGE) project. It aligns with ERGA’s objectives of fostering cooperation among European countries to develop genomic resources for biodiversity protection ( Mazzoni et al., 2023), as well as with the Portuguese coalition for biodiversity genomics initiative ( Marques et al., 2024).

## Materials & Methods

### Sample and sampling information

On 29th January 2024, an adult, female sample of *Patella rustica* was sampled by Rui Faria and Fernando P. Lima. The species was identified by Fernando Lima via morphology and color pattern. The specimen was dislodged with a knife from an intertidal rock and collected by hand in Porto, Portugal. Ethical sampling permits are not required for sampling of molluscs at this location. After dissection and sexing (based on gonad colouration - see Zegaoula *et al.* 2016 and Prusina *et al.* 2014) by Rocío Nieto Vilela, the specimen’s tissues (mollusc foot, gonad, head, hepatopancreas and other somatic tissue) were snap-frozen immediately in liquid nitrogen and stored at -80°C until they were shipped to CNAG, Barcelona in dry ice for subsequent laboratory procedures.

### Vouchering information

Shells for the here sequenced specimen and proxy specimen have been deposited in the Natural History and Science Museum of the University of Porto (MHNC-UP https://mhnc.up.pt/), under the accession numbers MHNCUP-MOL-24647 and MHNCUP-MOL-24646.

Frozen reference tissue material of muscle is available from the same and proxy individuals at the Museum Koenig Bonn ( https://bonn.leibniz-lib.de/de/) under the voucher IDs ZFMK-TIS:89067 and ZFMK-TIS-89068.

### Data availability

*Patella rustica* and the related genomic study were assigned to Tree of Life ID (ToLID) ‘xgPatRust1’ and all sample, sequence, and assembly information are available under the umbrella BioProject PRJEB84018. The sample information is available at the following BioSample accessions: SAMEA117648698, SAMEA115406481, SAMEA115406496 and SAMEA117648698. The genome assembly is accessible from ENA under accession number GCA_965111895.1 and the annotated genome is available through the Ensembl Beta site ( https://beta.ensembl.org/) and BGE Project Page ( https://projects.ensembl.org/erga-bge/). Sequencing data produced as part of this project are available from ENA at the following accessions: ERX13508601, ERX13629331, ERX13629332 and ERX13629333. Documentation related to the genome assembly and curation can be found in the ERGA Assembly Report (EAR) document available at https://github.com/ERGA-consortium/EARs/tree/main/Assembly_Reports/Patella_rustica/xgPatRust1. Further details and data about the project are hosted on the ERGA portal at https://portal.erga-biodiversity.eu/data_portal/87964.

### Genetic information

The estimated genome size, based on measurements across the Lottioidea superfamily ranges from 421Mb - 919Mb ( Hinegardner, 1974), while the estimation of the haploid genome size based on reads kmer profiling is 726 Mb. This is a diploid genome with a haploid number of 9 chromosomes (2n=18). Information for this species was retrieved from Genomes on a Tree ( Challis et al., 2023).

### DNA/RNA processing

DNA was extracted from the mollusc foot using the Blood & Cell Culture DNA Mini Kit (Qiagen) following the manufacturer’s instructions. DNA quantification was performed using a Qubit dsDNA BR Assay Kit (Thermo Fisher Scientific), and DNA integrity was assessed using a Femtopulse system (Genomic DNA 165 Kb Kit, Agilent). DNA was stored at 4°C until use.

RNA was extracted using an RNeasy Mini Kit (Qiagen) according to the manufacturer’s instructions. RNA was extracted from two different specimen body parts: mollusc foot, gonad and other somatic tissue. RNA quantification was performed using the Qubit RNA BR Kit and RNA integrity was assessed using a Fragment Analyzer system (RNA 15nt Kit, Agilent). RNA was stored at -80°C until use.

### Library preparation and sequencing

A long-read whole genome library was prepared using the SQK-LSK114 kit and sequenced on a PromethION P24 A series instrument (Oxford Nanopore Technologies). For short-read whole genome sequencing (WGS), a library was prepared using the KAPA Hyper Prep Kit (Roche). A Hi-C library preparation, using mollusc foot tissue, was conducted with the Dovetail Omni-C Kit (Cantata Bio) and further processed with the KAPA Hyper Prep Kit for Illumina sequencing (Roche). The RNA library, generated from mollusc foot, gonad and other somatic tissue, was prepared with the KAPA mRNA Hyper Prep Kit (Roche). All the short-read libraries were sequenced on the Illumina NovaSeq 6000 instrument. In total, 59x Oxford Nanopore, 65x Illumina WGS shotgun, and 147x Hi-C data were sequenced to generate the assembly.

### Genome assembly methods

The genome was assembled using the CNAG CLAWS pipeline ( Gomez-Garrido, 2024). Briefly, reads were preprocessed for quality and length using Trim Galore v0.6.7 and Filtlong v0.2.1, and initial contigs were assembled using NextDenovo v2.5.0, followed by polishing of the assembled contigs using HyPo v1.0.3, removal of retained haplotigs using purge-dups v1.2.6 and scaffolding with YaHS v1.2a. Finally, assembled scaffolds were curated via manual inspection using Pretext v0.2.5 with the Rapid Curation Toolkit ( https://gitlab.com/wtsi-grit/rapid-curation ) to remove any false joins and incorporate any sequences not automatically scaffolded into their respective locations in the chromosomal pseudomolecules (or super-scaffolds). Summary analysis of the released assembly was performed using the ERGA-BGE Genome Report ASM Galaxy workflow ( De Panis, 2024a), incorporating tools such as BUSCO v5.5 and Merqury v1.3. NextDenovo was chosen over the assembler Flye as it generated much better contiguity, with a contig N50 of 8.1Mb after the initial assembly, vs 301kb contig N50 generated by Flye. Flye also generated a contig assembly with a high proportion of detected duplicated BUSCO genes (29.1%), compared to the 1.8% found in the initial NextDenovo contig assembly.

### Genome annotation methods

A gene set was generated using the Ensembl Gene Annotation system ( Aken et al., 2016), primarily by aligning publicly available short-read RNA-seq data from BioSample: SAMEA117648698 to the genome. Gaps in the annotation were filled via protein-to-genome alignments of a select set of vertebrate proteins from UniProt ( The UniProt Consortium, 2019), which had experimental evidence at the protein or transcript level. At each locus, data were aggregated and consolidated, prioritising models derived from RNA-seq data, resulting in a final set of gene models and associated non-redundant transcript sets. To distinguish true isoforms from fragments, the likelihood of each open reading frame (ORF) was evaluated against known vertebrate proteins. Low-quality transcript models, such as those showing evidence of fragmented ORFs, were removed. In cases where RNA-seq data were fragmented or absent, homology data were prioritised, favouring longer transcripts with strong intron support from short-read data. The resulting gene models were classified into three categories: protein-coding, pseudogene, and long non-coding. Models with hits to known proteins and few structural abnormalities were classified as protein-coding. Models with hits to known proteins but displaying abnormalities, such as the absence of a start codon, non-canonical splicing, unusually small intron structures (<75 bp), or excessive repeat coverage, were reclassified as pseudogenes. Single-exon models with a corresponding multi-exon copy elsewhere in the genome were classified as processed (retrotransposed) pseudogenes. Models that did not fit any of the previously described categories did not overlap protein-coding genes, and were constructed from transcriptomic data were considered potential lncRNAs. Potential lncRNAs were further filtered to remove single-exon loci due to their unreliability. Putative miRNAs were predicted by performing a BLAST search of miRBase ( Kozomara et al., 2019) against the genome, followed by RNAfold analysis ( Gruber et al., 2008). Other small non-coding loci were identified by scanning the genome with Rfam ( Kalvari et al., 2018) and passing the results through Infernal ( Nawrocki & Eddy, 2013). Summary analysis of the released annotation was performed using the ERGA-BGE Genome Report ANNOT Galaxy workflow ( De Panis, 2024b), incorporating tools such as AGAT v1.2, BUSCO v5.5 and OMArk v0.3.

## Results

### Genome assembly

**Figure 2.**
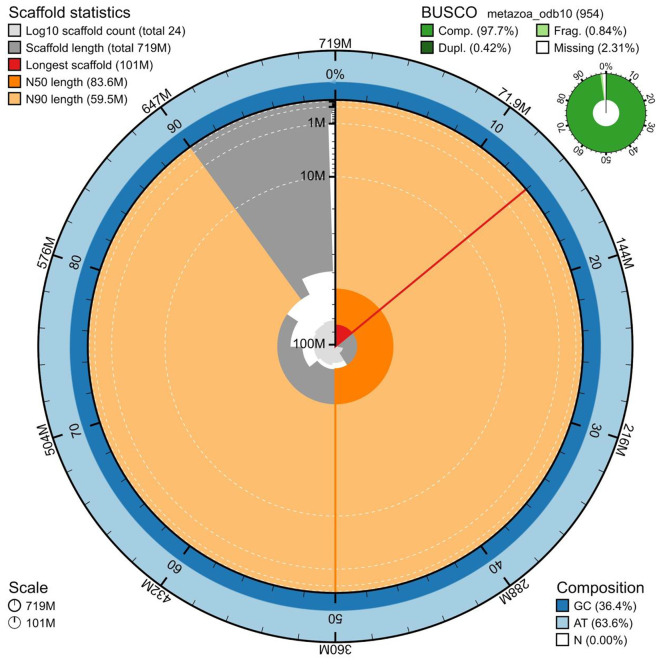
Snail plot summary of assembly statistics. The main plot is divided into 1,000 size-ordered bins around the circumference, with each bin representing 0.1% of the 719,386,390 bp assembly including the mitochondrial genome. The distribution of sequence lengths is shown in dark grey, with the plot radius scaled to the longest sequence present in the assembly (719,386,390 bp, shown in red). Orange and pale-orange arcs show the scaffold N50 and N90 sequence lengths 83,582,575 and 59,523,780 bp), respectively. The pale grey spiral shows the cumulative sequence count on a log-scale, with white scale lines showing successive orders of magnitude. The blue and pale-blue area around the outside of the plot shows the distribution of GC, AT, and N percentages in the same bins as the inner plot. A summary of complete, fragmented, duplicated, and missing BUSCO genes found in the assembled genome from the metazoa database (odb10) is shown on the top right.

**Figure 3.**
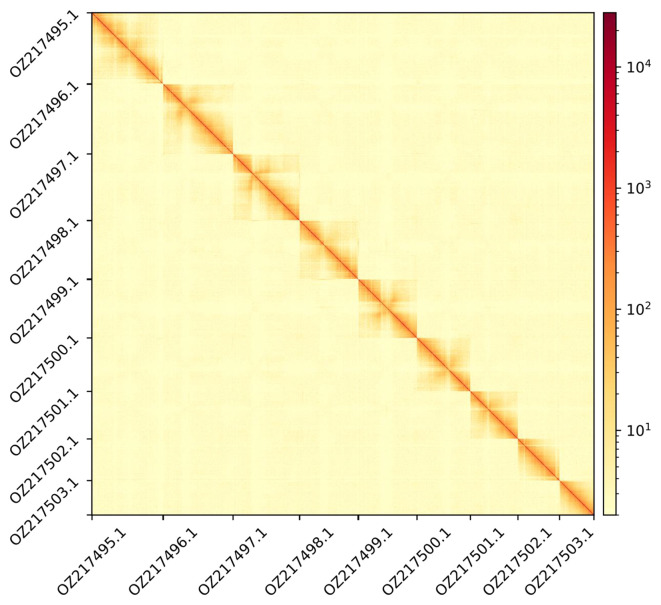
Hi-C contact map showing spatial interactions between regions of the genome. The diagonal corresponds to intra-chromosomal contacts, depicting chromosome boundaries. The frequency of contacts is shown on a logarithmic heatmap scale. Hi-C matrix bins were merged into a 400 kb bin size for plotting.

The genome assembly has a total length of 719,386,390 bp in 24 scaffolds including the mitogenome ( Figures 2 and 3), with a GC content of 36.4%. It features a contig N50 of 8,519,583 bp (L50=22) and a scaffold N50 of 83,582,575 bp (L50=4). Purge-dups removed 133 sequences identified as retained haplotigs, error or repeat sequences totalling 70Mb. This further reduced the proportion of duplicated BUSCO genes detected from 1.8% to 0.3%. There are 133 gaps, totaling 26.6 kb in cumulative size. The single-copy gene content analysis using the metazoa_odb10 database with BUSCO, run in genome assembly mode, resulted in 97.7% completeness (97.3% single and 0.4% duplicated). 81.41% of reads k-mers were present in the assembly and the assembly has a base accuracy Quality Value (QV) of 43.04 as calculated by Merqury. Overall 99.6% of the assembly was scaffolded into the 9 pseudo-chromosomal molecules.

### Genome annotation

The genome annotation consists of 20,636 protein-coding genes with an associated 37,053 transcripts, in addition to 22,234 non-coding RNA genes of various types ( Table 1). Using the longest isoform per transcript, the single-copy gene content analysis using the metazoa_odb10 database with BUSCO, run in protein mode, resulted in 93.2% completeness. Using the OMAmer Lophotrochozoa database for OMArk resulted in 95.91% completeness and 65.19% consistency ( Table 2).

**Table 1.** Statistics from assembled gene models.

	No. genes	No. transcripts	Mean * gene length (bp)	No. single-exon genes	Mean * exons per transcript
**Protein-coding **	20,636	37,053	18,629	834	8.9
**lncRNA**	13,909	19,144	16,846	5	2.2
**snRNA**	279	279	140	43	1.0
**snoRNA**	43	43	142	43	1.0
**rRNA**	1,120	1,120	221	1,120	1.0
**tRNA**	2,579	2,579	78	2,579	1.9
**scRNA**	1	1	133	1	1.0
**Other non-coding **	4,303	4,303	58-77	4,303	1.0

* Combined categories show the range of the mean values.

**Table 2.** Annotation completeness and consistency scores calculated by BUSCO run in protein mode (metazoa_odb) and OMArk (Lophotrochozoa).

	Complete	Singular	Duplicated	Fragmented	Missing
**BUSCO**	921 (96.5%)	915 (95.9%)	6 (0.6%)	9 (0.9%)	24 (2.5%)
**OMArk**	2,065 (95.91%)	1,918 (89.08%)	147 (6.83%)	-	88 (4.09%)
	**Consistent**	**Inconsistent**	**Contaminants**	**Unknown**	
**OMArk**	13,451 (65.18%)	1,914 (9.28%)	0 (0.00%)	5,271 (25.54%)	

### Author contributions

RF coordinated the project, RF and FL collected the species, FL and RNV identified the species, RF, FL and RNV sampled and preserved biological material and provided metadata, RF, RM, AB, TM, TS and RO provided sampling and metadata support and management, LA and MG extracted DNA, prepared libraries, and performed sequencing, FCC, JG-G and FC performed genome assembly and curation under the supervision of TA, LH and FM performed genome annotation, TB generated the analysis and report. All authors contributed to the writing, review, and editing of this genome note and read and approved the final version.
