# Supplementary material for: ERGA-BGE Chromosome-Level Genome of Patella rustica Linnaeus, 1758: a Resource to Investigate Responses to Global Warming in the intertidal
Source: Open Res Eur. 2026 Mar 9;5:350. Originally published 2025 Nov 11. [Version 2] doi: 10.12688/openreseurope.21608.2 (PMC12982983; doi:10.12688/openreseurope.21608.2)
Supplement: Peer review history

# Reviewer report: Reviewer response for version 2

**DOI:** 10.21956/openreseurope.24968.r70969

**Published:** 2026-03-12

**Authors:** Craig Wilding

**Affiliations:** 1 University College Dublin, Dublin, Ireland

The authors present a high-quality chromosomally anchored reference genome for *Patella rustica *which will, as the authors state in the introduction, provide a valuable resource for both empowering population genomics in this species, but also facilitate comparative genomic analysis within the limpets.

 I have only minor comments:

 Materials and methods:

 I thought the sub-heading 'Genetic information' to be unhelpful since this section deals only with genome size estimation, and perhaps therefore this is a more useful heading. The first sentence of this section "The estimated genome size, based on measurements across the Lottioidea superfamily ranges from 421Mb - 919Mb (Hinegardner, 1974)" could perhaps be more clearly worded since, presumably, this large range in values is not the estimated genome size of this species. Perhaps it could be worded more clearly as "There is no published estimate of genome size for *Patella rustica*, but measurements across the Lottioidea superfamily range from 421Mb - 919Mb (Hinegardner, 1974)"

 DNA/RNA processing section: "RNA was extracted from two different specimen body parts: mollusc foot, gonad and other somatic tissue." Isn't this three different body parts?

 Results:

 Genome annotation section - needs a citation for "OMAmer Lophotrochozoa database for OMArk" both here, and in the methods.

 Table 1 - please be clear on that "Mean * gene length" includes introns.

Are sufficient details of methods and materials provided to allow replication by others?

Yes

Is the rationale for creating the dataset(s) clearly described?

Yes

Are the datasets clearly presented in a useable and accessible format?

Yes

Are the protocols appropriate and is the work technically sound?

Yes

Reviewer Expertise:

Evolutionary genetics

I confirm that I have read this submission and believe that I have an appropriate level of expertise to confirm that it is of an acceptable scientific standard.

# Reviewer report: Reviewer response for version 1

**DOI:** 10.21956/openreseurope.23376.r64049

**Published:** 2025-12-11

**Authors:** Yasuto ISHII

**Affiliations:** 1 Tohoku University, Sendai, Japan

**GENERAL COMMENTS**

 The present study released a genome assembly of *Patella rustica*. The assembly have high continuity and completeness, so thereby it should be valuable genetic resource. Also, the authors provided a high-quality gene annotation which is also a promising genetic resource. The assembly and annotation are available from a public database. Collectively, the assembly and annotation should promote further genomic research in *Patella *snails and other related species. Yet, this manuscript does not contain fundamental information, such as details of pre-Hi-C scaffolding assembly and how much sequences were anchored on the Hi-C scaffolds. Also, some fruitful analyses, including repeat masking, are lack in the current version. I listed some minor comments and suggestions below.

**COMMENTS**

 Title and abstract: ERGA-BGE is not a common abbreviation. The full name should be addressed in the abstract. Also, I suggest that the word "chromosome-level assembly" should be included in the title to be appealing to readers. If the word limit is not strict, sequencing methods (e.g., the use of long-read, read type [ONT+Hi-C+short-read]) and BUSCO values should be noted in the abstract. This information ensures the validity of the assembly. Furthermore, the taxonomic information (e.g., family, class; I’m not familiar with limpets, so not sure what rank is useful) is helpful for readers to reach this article.

 In the introduction, while the authors stated that the species is serve as a "sentinel", the reason why this species particularly has such a character.

 Add appropriate reference(s) for the following sentence: "Several studies have documented recent changes in its distribution, particularly the bridging of a historical distribution gap in northwestern Iberia."

 How did you detect the sex?: "female sample of *Patella rustica* was sampled"

 I suggest that you should do the genome size estimation by yourself. In my opinion, articles should be self-contained if possible.: "the estimation based on reads kmer profiling is 726 Mb"

 I cannot grasp the meaning of this sentence. Does it mean that the estimation is for the haploid genome?: "This is a diploid genome with a haploid number of 9 chromosomes (2n=18) "

 To be clear, did you perform genome annotation without repeat masking? One intriguing feature of molluscan genomes are the evolution of repeat elements. If you can do it, it should be a valuable resource. Also, while I’m not familiar with the gene annotation pipeline, generally repeat masking improves the quality of gene annotations.

 In Table 2, you reported the proportion of contaminations is 0.05 (lack of "%"?) but the number of contaminated transcripts is zero. It looks weird. The proportion should be 0.00%; otherwise, the number of contaminated transcripts is wrong.

 More details of the assembly should be reported, including the statistics of the assembly before Hi-C scaffolding. Moreover, according to Fig. 3, the sequences look to be anchored on nine mega-scaffolds, but it is not clear due to the lack of the mention in the main text. If so, the number of mega-scaffolds is matched with the haploid number of chromosomes of this species (documented in the method section); this information is noteworthy. It is also unclear how much sequences are anchored on the mega-scaffolds.

Are sufficient details of methods and materials provided to allow replication by others?

Yes

Is the rationale for creating the dataset(s) clearly described?

Partly

Are the datasets clearly presented in a useable and accessible format?

Yes

Are the protocols appropriate and is the work technically sound?

Yes

Reviewer Expertise:

Genomics on molluscs

I confirm that I have read this submission and believe that I have an appropriate level of expertise to confirm that it is of an acceptable scientific standard, however I have significant reservations, as outlined above.

# Response

**Published:** 2026-02-03

**Authors:** Thomas Brown

**Affiliations:** Evolutionary Genetics, Leibniz Institute for Zoo- and Wildlife Research, Berlin, Berlin, Germany

**GENERAL COMMENTS** The present study released a genome assembly of Patella rustica. The assembly have high continuity and completeness, so thereby it should be valuable genetic resource. Also, the authors provided a high-quality gene annotation which is also a promising genetic resource. The assembly and annotation are available from a public database. Collectively, the assembly and annotation should promote further genomic research in Patella snails and other related species. Yet, this manuscript does not contain fundamental information, such as details of pre-Hi-C scaffolding assembly and how much sequences were anchored on the Hi-C scaffolds. Also, some fruitful analyses, including repeat masking, are lack in the current version. I listed some minor comments and suggestions below.

**Thank you for the suggestions. We have added to the text following the guidelines of the reviewer:**

**COMMENTS**

**Title and abstract: ERGA-BGE is not a common abbreviation. The full name should be addressed in the abstract. Also, I suggest that the word "chromosome-level assembly" should be included in the title to be appealing to readers. If the word limit is not strict, sequencing methods (e.g., the use of long-read, read type [ONT+Hi-C+short-read]) and BUSCO values should be noted in the abstract. This information ensures the validity of the assembly. Furthermore, the taxonomic information (e.g., family, class; I’m not familiar with limpets, so not sure what rank is useful) is helpful for readers to reach this article.**

 Thank you for the suggestions. We have added “Chromosome-Level” to the title, further details of the class and family (Gastropoda, Patellidae), as well as the sequencing recipe and the project and consortia to the abstract.   

**In the introduction, while the authors stated that the species is serve as a "sentinel", the reason why this species particularly has such a character.**

 We would like to clarify that this is not a particular attribute of our focal species and that the term was not coined by us. We only use it in the introduction to refer to other intertidal species, in the sense that they can inform us about environmental change, for instance, via shifts in their distribution ranges as a consequence of global warming (as mentioned in the introduction and supported by references). We hope that this is clear in the main text and no further changes are needed.  

**Add appropriate reference(s) for the following sentence: "Several studies have documented recent changes in its distribution, particularly the bridging of a historical distribution gap in northwestern Iberia.**

 Thank you for the suggestion. We added “(Fischer-Piètte and Gaillard, 1959; Lima et al., 2006)” at the end of the sentence. And added the first reference to the final list.  

**How did you detect the sex?: "female sample of Patella rustica was sampled"**

 The sex of the individual was determined macroscopically as they are easily distinguishable by gonad coloration: females have green or brown gonads, while males present a pinkish-cream color (based on previous work describing the reproductive cycles of the species, with some examples in Zegaoula 2016 and Prusina 2014). We added the following information in one of the subsequent sections of the same section with the new text underlined:  After dissection and sexing (based on gonad colouration) by Rocío Nieto Vilela, the specimen's tissues (mollusc foot, gonad, head, hepatopancreas and other somatic tissue)    Refs Zegaoula, B., Beldi, H., Draredja, B., & Soltani, N. (2016). Reproduction of Patella rustica (Mollusca, Gastropoda) in the gulf of Annaba (Algeria, Mediterranean South Western). Advances in Environmental Biology, 10(11), 42-51. Prusina, I., Ezgeta-Balić, D., Ljubimir, S., Dobroslavić, T., & Glamuzina, B. (2014). On the reproduction of the Mediterranean keystone limpet Patella rustica: histological overview. Journal of the Marine Biological Association of the United Kingdom, 94(8), 1651-1660.  

**I suggest that you should do the genome size estimation by yourself. In my opinion, articles should be self-contained if possible.: "the estimation based on reads kmer profiling is 726 Mb"**

 We performed genome size estimation using the sequenced genomic DNA, estimating the size based on total coverage and ratio of heterozygous - homozygous kmers. To add context in the manuscript, we have added the range of genome sizes identified by Hinegardner, however performing genome size calculation from scratch is not within the scope of this study.  

**I cannot grasp the meaning of this sentence. Does it mean that the estimation is for the haploid genome?: "This is a diploid genome with a haploid number of 9 chromosomes (2n=18)** "

 We apologise for the confusion and have explicitly stated that the size estimates correspond to the haploid genome size.  

**To be clear, did you perform genome annotation without repeat masking? One intriguing feature of molluscan genomes are the evolution of repeat elements. If you can do it, it should be a valuable resource. Also, while I’m not familiar with the gene annotation pipeline, generally repeat masking improves the quality of gene annotations.**

 The gene annotation pipeline used did not make use of masked repetitive elements, however soft- and hard-masked versions of the genome assembly are available via the Ensembl download site alongside the annotation files.  

**In Table 2, you reported the proportion of contaminations is 0.05 (lack of "%"?) but the number of contaminated transcripts is zero. It looks weird. The proportion should be 0.00%; otherwise, the number of contaminated transcripts is wrong.**

 Thank you for noticing our typo - which came from 5 and % sharing a key - this has been corrected in the table.  

**More details of the assembly should be reported, including the statistics of the assembly before Hi-C scaffolding. Moreover, according to Fig. 3, the sequences look to be anchored on nine mega-scaffolds, but it is not clear due to the lack of the mention in the main text. If so, the number of mega-scaffolds is matched with the haploid number of chromosomes of this species (documented in the method section); this information is noteworthy. It is also unclear how much sequences are anchored on the mega-scaffolds.**

 We have added details as to the choice of assembler in the methods section and the amount of sequence removed by the purge-dups step in creating the assembly for a single haplotype. We have furthermore added the total proportion of the assembly anchored to the pseudo-chromosomal molecules to the results section.

# Reviewer report: Reviewer response for version 1

**DOI:** 10.21956/openreseurope.23376.r64050

**Published:** 2025-11-24

**Authors:** Jacob A Tennessen

**Affiliations:** 1 Harvard T. H. Chan School of Public Health, Boston, Massachusetts, USA

This is a nicely assembled and annotated genome that will be a useful resource for the mollusk genomics community. Assembly and annotation methods are appropriately described, albeit with minimal details.

 The genome is from an outbred diploid individual, and it would be nice to see more details on how heterozygous sites and more complex structural polymorphisms were handled. Presumably they were arbitrarily collapsed and it says haplotigs were removed. Is there an estimate of genome-wide heterozygosity (e.g. from alignment of the short reads to the final assembly)? Is it possible that some contigs/scaffolds are allelic copies of each other?

 The authors mention the genomes of five other *Patella* species. These should be cited in this paper (if no publication exists, then link to their accessions on ENA or https://beta.ensembl.org). The utility of this genome will largely be found in the context of those congener genomes: comparative work will allow errors to be identified and corrected, while also revealing informative evolutionary patterns. It would be interesting to see such comparative work included here, though I understand if such analysis is beyond the scope of the funded project.

 Change (Hoffmann, 2011) to (Hoffmann & Sgrò, 2011)

Are sufficient details of methods and materials provided to allow replication by others?

Yes

Is the rationale for creating the dataset(s) clearly described?

Yes

Are the datasets clearly presented in a useable and accessible format?

Yes

Are the protocols appropriate and is the work technically sound?

Yes

Reviewer Expertise:

Evolutionary genetics, including genomics of snails

I confirm that I have read this submission and believe that I have an appropriate level of expertise to confirm that it is of an acceptable scientific standard.

# Response

**Published:** 2026-02-03

**Authors:** Thomas Brown

**Affiliations:** Evolutionary Genetics, Leibniz Institute for Zoo- and Wildlife Research, Berlin, Berlin, Germany

**Review: **This is a nicely assembled and annotated genome that will be a useful resource for the mollusk genomics community. Assembly and annotation methods are appropriately described, albeit with minimal details.   The genome is from an outbred diploid individual, and it would be nice to see more details on how heterozygous sites and more complex structural polymorphisms were handled. Presumably they were arbitrarily collapsed and it says haplotigs were removed. Is there an estimate of genome-wide heterozygosity (e.g. from alignment of the short reads to the final assembly)? Is it possible that some contigs/scaffolds are allelic copies of each other? As much as possible, any retained sequences from the other haplotype were removed both during the purge-dups and manual curation steps. An analysis of any heterozygosity levels or regions is not considered part of this data note.   The authors mention the genomes of five other Patella species. These should be cited in this paper (if no publication exists, then link to their accessions on ENA or https://beta.ensembl.org). The utility of this genome will largely be found in the context of those congener genomes: comparative work will allow errors to be identified and corrected, while also revealing informative evolutionary patterns. It would be interesting to see such comparative work included here, though I understand if such analysis is beyond the scope of the funded project.

**Response: **Thank you for the suggestion. We have included the citations for 4 papers where the genomes have been published in a corresponding article. For the 5th genome, we have included the genbank accession.   Change (Hoffmann, 2011) to (Hoffmann & Sgrò, 2011) Thank you for noticing the error - we have corrected the citation in the text
